# Quadricuspid Pulmonary Valve With Fenestration: Cadaveric Findings

**DOI:** 10.7759/cureus.42705

**Published:** 2023-07-30

**Authors:** Graham Blair, Juan J Cardona, Arada Chaiyamoon, Devendra Shekhawat, Joe Iwanaga, Marios Loukas, R. Shane Tubbs

**Affiliations:** 1 Anatomy, Tulane University School of Medicine, New Orleans, USA; 2 Department of Neurosurgery, Tulane University School of Medicine Center for Clinical Neurosciences, New Orleans, USA; 3 Department of Anatomy, Faculty of Medicine, Khon Kaen University, Khon Kaen, THA; 4 Department of Oral and Maxillofacial Anatomy, Graduate School of Medical and Dental Sciences, Tokyo Medical and Dental University, Tokyo, JPN; 5 Department of Structural and Cellular Biology, Tulane University School of Medicine, New Orleans, USA; 6 Department of Neurology, Tulane University School of Medicine, New Orleans, USA; 7 Department of Anatomical Sciences, St. George’s University, St. George’s, GRD; 8 Department of Neurosurgery, Ochsner Neuroscience Institute, Ochsner Health System, New Orleans, USA; 9 Department of Surgery, Tulane University School of Medicine, New Orleans, USA

**Keywords:** clinical, anatomy, congenital cardiac disease, anatomical variation, pulmonary trunk, quadracuspid pulmonary valve

## Abstract

Quadricuspid pulmonary valves (QPV) are rare entities. Such valves can be associated with other cardiac anatomical anomalies. In this report, we present a case of a quadricuspid valve with an additional variant and discuss the morphometrics of this anatomical variation. During the routine dissection of an adult male body, two anatomical variations were found within the pulmonary trunk. This individual had a QPV. In addition, one of the leaflets of this valve contained fenestrations. No additional cardiac anomalies were identified. Clinicians who review imaging of the heart or treat patients with cardiac conditions should be well-informed about QPV.

## Introduction

While bicuspid semilunar valves receive more attention in the medical literature due to their propensity to stenose, quadricuspid or quadrivalent pulmonary valves (QPVs) are also anatomical irregularities that physicians have been familiar with for a long time; Leonardo da Vinci, in fact, included both bicuspid and quadricuspid sketches of valves alongside the more common three-leaflet variety in the 16th century based on the bodies he evaluated [1]. Previously, it was hard to ascertain actual incidence rates of supernumerary aortic and pulmonary valves because they are often completely functional or subclinical; however, with the availability of improved echocardiography techniques these days, studying noncomplicated patients is more feasible, and measured incidence rates are becoming more accurate [2]. Current estimates of the incidence of QPVs range from 0.1 to 0.2% of the general population [3,4]. While Hurwitz type-b QPVs are the most common variant, Hurwitz type-a cases are more often clinically diagnosed and more commonly associated with pulmonary regurgitation [5]. This contrasts with quadricuspid aortic valves (QAVs), which, while rarer, are insufficient valves about half of the time [6].

We adhered to all laws and guidelines pertaining to the use of human bodies for research [7] while conducting this study.

## Case presentation

**Figure 1:**
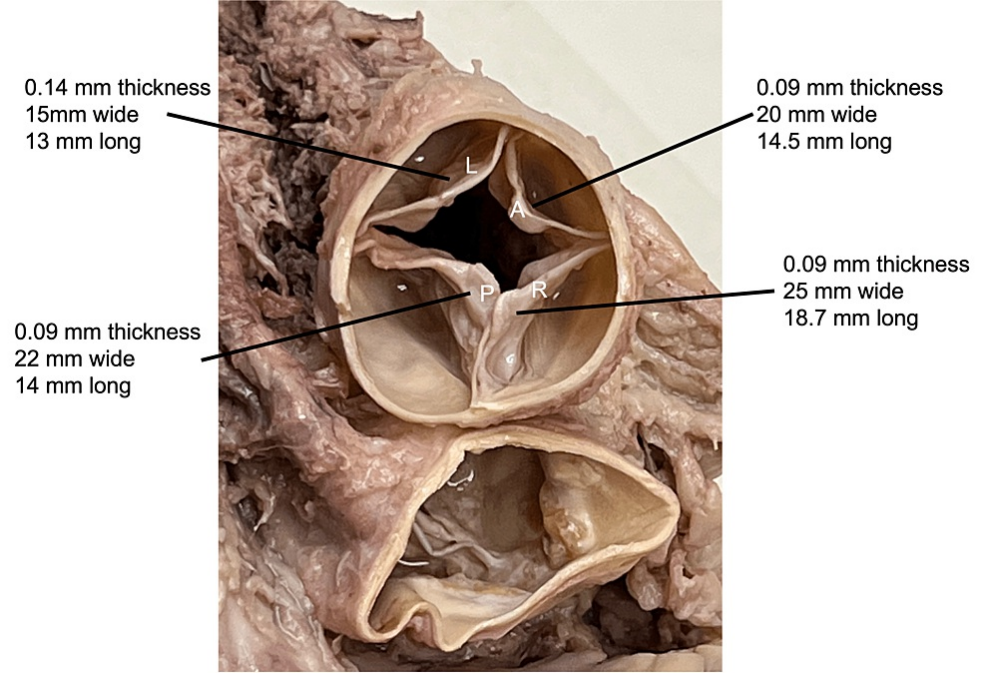
Superior view of the internal aspect of the pulmonary trunk showing the four semilunar leaflets and the measurements for each of these L: left leaflet; A: anterior leaflet; R: right leaflet; P: posterior leaflet

**Figure 2:**
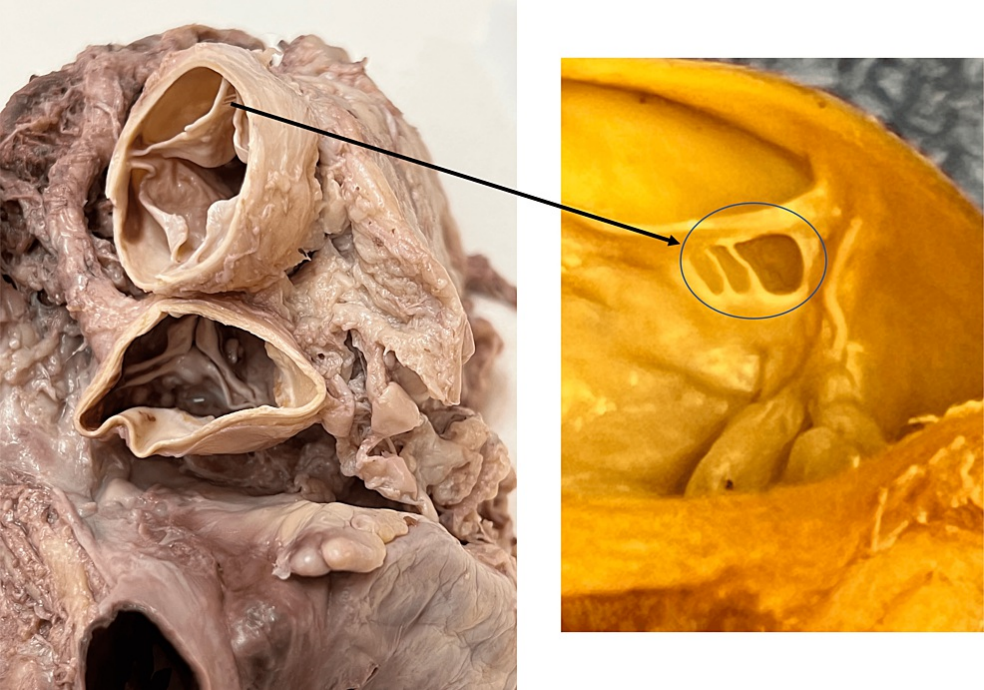
Anterosuperior (left) and zoomed-in (right) views of the fenestrated pulmonary valve leaflet

During the routine dissection of the heart in an 87-year-old male body, an unusual finding of the pulmonary valve was observed. The individual was formalin-fixed and had died of natural causes. There had been no known history of cardiac pathology. After removing the heart from the thorax, four pulmonary valve leaflets were found. Specifically, a posterior leaflet was present (Figure 1). The arrangement and measurements of each leaflet are seen in Figures 1, 2.

As one leaflet (left leaflet) in this individual was slightly smaller than the others, it was classified as a Hurwtiz type-b valve. Additionally, one of the leaflets (left leaflet) was found to be fenestrated (Figure 2). These fenestrations were found in the anterior-most aspect of the valve at its attachment to the internal surface of the pulmonary trunk. The fenestration (the entire defect in the leaflet and including each of the smaller windows) roughly measured 5 x 3 mm. The length of the leaflets was made where the length was along the free edge and the width was the maximal length located at the leaflet’s midpoint. The remaining visible morphology of the heart was found to be within normal limits, including some mild calcifications within the ascending aorta and left and right coronary arteries. No hypertrophy of the right ventricle or dilatation of the pulmonary trunk or conus arteriosus was found in relation to the pulmonary trunk and QPV. All the measurements were made with microcalipers (Mitutoyo, Kawasaki, Japan).

## Discussion

While the pulmonary valve normally has three semilunar leaflets (left, right, and anterior), in some cases, it can have an abnormal number of leaflets, such as two, four, or more (only one case of pentacuspid valve has been reported in the literature to date) [8,9]. Recently, Lis et al. [5] revisited the anatomy of the tricuspid pulmonary valve and provided several morphometrical and geometrical descriptions. For instance, they reported that the mean intercommissural distance and geometric height (width and length in our study respectively) of the left anterior, right anterior, and posterior leaflets were 17.36 ±4.25 - 15.25 ±3.10 mm, 17.21 ±4.27 - 15.49 ±2.79 mm, and 17.62 ±3.61 - 15.69 ±3.38 mm respectively. Also, the authors identified the presence of fenestrations in all the leaflets of the pulmonary valve, with an occurrence rate of 12.5% [5].

QPVs can be further classified based on the sizes of the four leaflets: ~60% have three similarly sized leaflets with one smaller leaflet (Hurwitz type-b); ~15% have two similarly sized larger leaflets and two similarly sized smaller leaflets (Hurwitz type-c); ~12% have four equally sized leaflets (Hurwitz type-a); and the rest of the cases have various other dimensions regarding leaflets’ sizes (Hurwitz types d-g) [3,10]. Solewski et al. [9] documented the histomorphological analysis of a QPV found in a 26-year-old male. They found that the leaflet length and height (width and length in our study respectively) of the left anterior, right anterior, posterior, and the additional leaflet were 14.6 - 15.0 mm, 13.5 - 11.6 mm, 13.3 - 13.6 mm, and 5.2 - 10.1 mm respectively. Additionally, in this study, the thickness of the tissue was measured in selected valve regions (distal part of the leaflet - left: 0.28 mm, right: 0.28 mm, posterior: 0.29 mm, additional: 0.24 mm; proximal part of the leaflet - left: 0.78 mm, right: 0.49 mm, posterior: 0.46 mm, additional: 0.52 mm), obtaining slightly larger measurements in comparison with ours [9].

The supernumerary leaflets are generally asymptomatic and isolated; also, they can rarely cause dysfunction and are more commonly associated with other congenital cardiac abnormalities [11]. Interestingly, combining data from two studies, Hurwitz et al.'s [10] and Davia et al.'s [6], of the 193 QPVs found at necropsy, only eight (4%) were not fully functional [6]. Alternatively, QPVs can be associated with congenital cardiac abnormalities such as patent ductus arteriosus, atrial or ventricular septal defects, and bicuspid aortic valves [12]. In fact, compared with QAVs, QPVs are more often coexistent with congenital heart anomalies and valvular stenosis (but less frequently coexistent with coronary artery anomalies and infectious endocarditis) [11]. Because of its association with other cardiac abnormalities, understanding the embryology of valvulogenesis and how these abnormalities might arise is critical. Some alleles, such as epidermal growth factor receptor (Egfr) and protein tyrosine phosphatase for Shp2 protein (Ptpn11), have been demonstrated in animal models as important for only semilunar valvulogenesis, but not for atrioventricular valves [13]. Other animal studies have shown that bicuspid variations of the aortic valve result from the fusion of the valve cushion primordia and that the early existence of three valve primordia is the norm, even for bicuspid valves [14]. While this latter theory would perhaps explain why bicuspid semilunar valves are more common than quadricuspid valves, it does not in fact offer a theory for the formation of quadricuspid valves.

The cushions that give rise to the leaflets of the pulmonary valve are made up of neural crest cells, but the mechanism by which these cushions actually develop into the leaflets of the semilunar valves remains unresolved [15]. While it was previously thought that late gestation signaling was more involved in the neural crest cells’ role, a more recent study involving RockDN and Wnt1 mutants in mouse models suggests that earlier signaling of neural crest cells may be responsible for both bicuspid and quadricuspid valves [16]. By interfering with RockDN signaling, well-defined outflow cushions fail to form, or are misplaced: sometimes, the non-coronary leaflet does not form at all, and bicuspid valves appear to emerge; in other cases, neural crest cells aggregate inappropriately, producing extra cushions that may give rise to supernumerary valves [16]. Therefore, the organization of the neural crest cells may predict the semilunar valve structure, regardless of the type.

Declaration

The authors sincerely thank those who donated their bodies to science so that anatomical research could be performed. Results from such research can potentially increase mankind’s overall knowledge which can then improve patient care. Therefore, these donors and their families deserve our highest gratitude [17].

## Conclusions

QPVs tend to be asymptomatic and are commonly related to other congenital cardiac anomalies. It is vital for clinicians treating cardiac conditions or reviewing cardiac images to have adequate knowledge about this anatomical variation, which was also found in this case along with a fenestration in the left leaflet, and the embryological correlation discussed; also, this case is of archival value for future descriptions of these concurrent variations or similar cases.
